# Features of migraine aura in teenagers

**DOI:** 10.1186/1129-2377-15-87

**Published:** 2014-12-12

**Authors:** Igor Petrusic, Vera Pavlovski, Dragana Vucinic, Jasna Jancic

**Affiliations:** 1 Clinic of Neurology and Psychiatry for Children and Youth, CCS, Doktora Subotica 6a, 11000 Belgrade, Serbia; 2 Faculty of Medicine, University of Belgrade, Doktora Subotica 8, 11000 Belgrade, Serbia

**Keywords:** Migraine aura, Higher cortical dysfunction, Teenagers

## Abstract

**Background:**

Complex migraine aura in teenagers can be complicated to diagnose. The aim of this study was to present detailed features of migraine aura in teenage migraineurs.

**Methods:**

This cross-sectional study was conducted in the period from 2008 till 2013. A total number of 40 teenage migraineurs (20 females and 20 males) met criteria for this study. The patients were interviewed using a specially designed questionnaire for collecting data about migraine aura features. Main outcome measures were frequency of visual, somatosensory and higher cortical dysfunction (HCD) symptoms in teenage migraineurs population during the aura, and also within each individual.

**Results:**

Visual aura was reported in every attack, followed by somatosensory (60%) and dysphasic (36.4%) aura. Scintillating scotoma and blurry vision were mostly reported and predominant visual symptoms. The most common somatosensory symptom was numbness in hand. HCD were reported by 22 (55%) patients. Slowed speech was mostly reported symptom of HCD, followed by dyslexia, déjà vu phenomenon, color dysgnosia, and dyspraxia. In patients with HCD, aura frequency per year (6.18 ± 3.17 vs. 3.33 ± 2.03, p = 0.003) and prevalence of somatosensory symptoms (77.3% vs. 38.9%, p = 0.014) were significantly higher than in patients without HCD.

**Conclusions:**

Aura symptoms vary to a great extent in complexity in teenage migraineurs. Consequently, results obtained in this study provide useful information for clinicians when faced with unusual migraine aura.

## Background

The estimated overall mean prevalence of migraine in children and adolescents worldwide was 7-11% [1]. Thereof, 25% of patients with migraine experience an aura [2]. Migraine aura is commonly considered to precede headache [3]. Visual auras are the most common, followed by somatosensory, and then dysphasic auras [4]. Motor aura is the least common and is a defining feature of hemiplegic migraine [5].

Migraine aura is thought to arise due to a change in cortical neural excitability and function [6]. Cortical spreading depolarization followed by cortical spreading depression (CSD), mostly originates in the occipital region [7]. The involvement of other cortical areas beyond the occipital region could be assumed because of the existence of somatosensory and memory clinical features during the aura in some patients [8,9].

Diagnosis of migraine in the developmental age is more difficult and associated with imprecise description of the symptoms. Moreover, acute confusional migraine is primarily seen in children and adolescents [10]. The confusional state often manifests with a wide diversity of cortical dysfunctions, such as speech difficulties, amnesia, dysgnosia and dyspraxia [11]. Knowledge of migraine aura symptoms, clinical differences associated with developmental age and features are very important in differentiation with other disorders imitating migraine [12].

This study represents an attempt to present detailed features of migraine aura in teenagers. Furthermore, the aim of this study was to evaluate the frequency and types of higher cortical dysfunctions (HCD) that occur during the aura.

## Methods

Total of 67 teenagers having migraine with aura, treated from the beginning of 2008 to the end of 2013 (six years), at the Clinic of Neurology and Psychiatry for Children and Youth, Medical Faculty, University of Belgrade, were called to participate in this study. The diagnosis was based on the International Classification of Headache Disorders criteria [13]. Excluding criteria were: other neurological diseases, motor aura symptoms [13], chronic migraine and patients who did not respond to a call. Forty patients who met inclusion criteria have accepted to participate in this study. A special questionnaire (Table 1) was designed to collect data on migraine aura features and HCD during the aura. Positive responses to each question of designed questionnaire were followed by the sub-questions for the purpose of collecting information how long this symptom lasts, when this symptom starts in comparison to beginning of headache, how frequent this symptom is present in aura (in percentage) and whether symptom develops gradually. The patients were interviewed by a doctor (I.P. or V.P.), experienced in headache research. Research protocol of this study was approved by the review board of the Clinic of Neurology and Psychiatry for Children and Youth, Medical Faculty, University of Belgrade.

**Table 1:** Study questionnaire

During the aura of your migraine attack, have you ever noticed:	
1.	Shimmering or blurred dots in the visual field?
2.	Twinkling zig-zag lines in the visual field?
3.	Blurred vision (like looking through tick glass)?
4.	Tunnel vision (narrowing of the visual field)?
5.	Tingling or numbness in fingers, hand, leg, face (lips) and tongue?
6.	Changes in colors? Did colors get brighter or paler?
7.	Difficulties in recognizing faces, unrelated to the disturbance of vision?
8.	Difficulties in recalling names?
9.	Difficulties in recalling events from the past?
10.	Difficulties in remembering events during aura?
11.	The feeling that you have already seen events (déjà vu phenomenon)?
12.	Difficulties in speaking even when you knew what you wanted to say?
13.	Did someone tell you that you speaking gibberish?
14.	It takes more time to find the appropriate words when you try to speak?
15.	Difficulties in understanding speech or recognizing sounds from the environment?
16.	Difficulties in understanding writing, unrelated to visual disorders?
17.	Difficulties in writing that were not caused by the disturbance of vision?
18.	Difficulties in calculating and/or memorizing numbers?
19.	Difficulties in recognizing objects by touch?
20.	Difficulties in performing normal movements with your hands?
21.	Difficulties in orientation in space (in terms of left and right)?
22.	Unawareness of one part of your body?

The data are presented as arithmetic mean values ± SD or as percentages. For analysis purpose, we formed a group of patients who experienced one or more HCD symptoms during the aura (HCD group) and a group of patients who did not experience HCD (Standard aura group). Independent samples t test was used to compare the age of patients and the age at the time of the onset of migraine with aura; Chi squared test was used to compare gender, number of patients who reported somatosensory symptoms in general and who reported numbness in hand; Fisher's exact test was used to compare the number of patients who reported numbness of arm, leg, tongue, face and lips; and the Mann–Whitney U test was used to compare aura duration and the number of auras per year between the groups. The significance level for the analysis was set beforehand at 5% (p < 0.05).

## Result

The study included 20 females and 20 males, aged 16.2 ± 2.0 (range 13–19) years, who experienced migraine with aura. Fifteen (37.5%) patients had visual aura only; 10 (25.0%) patients had visual and somatosensory auras; and 15 (37.5%) patients had visual with/or without somatosensory and with dysphasic aura.

Visual and somatosensory symptoms of aura in teenage migraineurs are described in Table 2. All patients had one or more visual symptoms. Scintillating scotoma was the most commonly reported (67.5%) and predominant (94%) visual symptom during the aura. Somatosensory symptoms were less common than visual with occurrence of 60% in patients. Most common was numbness in the left hand or both hands (2 patients) reported in 21 patients (52.5%), followed by numbness in: lips and/or face (30%), tongue (27.5%), and legs (15%). Two patients had acute onset visual aura, followed by short presentation of somatosensory symptoms and HCD. Also, two patients had prolonged visual aura, who reported gradual developing of somatosensory and dysphasic symptoms. Overall, in ten patients migraine aura proceeded during headache for 12.6 ± 10.2 (range 3–30) minutes and four patients reported period of 7.5 ± 2.9 (range 5–10) minutes between migraine aura and onset of headache. Twenty-six patients reported onset of headache immediately after finish of aura.

**Table 2:** Visual and somatosensory symptoms during the aura

**Visual and somatosensory aura features**	**Number of patients**	**Onset time X ± SD (min-max)**	**Time duration X ± SD (min-max)**	**Frequency X ± SD (min-max)**
	**n = 40 (%)**			
Scintillating scotoma	27 (67.5)	21.24 ± 15.87 (25–60)	18.67 ± 16.62 (2–75)	94.26 ± 17.25 (25–100)
Zig-zag lines	10 (25)	23 ± 12.06 (5–45)	19.5 ± 12.12 (5–45)	90 ± 23.09 (30–100)
Blurry vision	24 (60)	22.22 ± 12.12 (2–45)	19.29 ± 14.53 (2–45)	85 ± 27.54 (10–100)
Tunnel vision	16 (40)	24.69 ± 14.54 (5–60)	18.44 ± 12.48 (5–60)	74.38 ± 35.02 (10–100)
Somatosensory symptoms	24 (60)	22.06 ± 17.3 (2–60)	17.5 ± 16.9 (2–60)	62.5 ± 31.21 (10–100)

Onset time - aura onset in regard to beginning of headache expressed in minutes; Time duration - expressed in minutes; Frequency - frequency of symptom compared to all experienced auras in individual (expressed in percentages).

**Figure 1:**
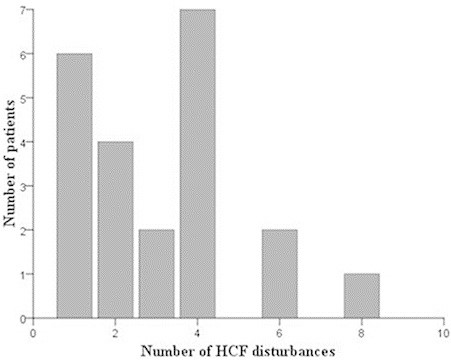
**Distribution of patients by number of HCF disturbances.** HCF: higher cortical function.

HCD were reported by 22 (55%) patients in this study. A detailed description of HCD during the aura in teenage migraineurs analyzed group (40 patients) was given in Table 3. The majority of patients with HCD during the aura reported one, two, or four HCD symptoms, as shown in Figure 1. Slowed speech was the most usually reported (27.5%) symptom of HCD during the aura, followed by dyslexia (25%), déjà vu phenomenon (22.5%), color dysgnosia (20%), and dyspraxia (20%). Prosopagnosia was reported in one (2.5%) patient, while difficulties in understanding speech or recognizing sounds and difficulties in writing were not reported. Color dysgnosia, slowed speech and manual dyspraxia were most frequently experienced symptoms. Moreover, 14 (35%) patients have experienced one or more HCD symptoms in more than one third of their auras.

**Table 3:** Features of HCD during the aura reported in teenage migraineurs

**HCD**	**Number of patients**	**Onset time X ± SD (min-max)**	**Time duration X ± SD (min-max)**	**Frequency X ± SD (min-max)**
	**n = 40 (%)**			
Color dysgnosia	8 (20)	8.5 ± 6.48 (2–20)	7.13 ± 6.1 (2–20)	60 ± 40.71 (10–100)
Dysnomia	3 (7.5)	15.67 ± 14.01 (2–30)	15.67 ± 14.01 (2–30)	46.67 ± 46.19 (20–100)
Retrograde amnesia	4 (10)	16.75 ± 15.35 (2–30)	16.75 ± 15.35 (2–30)	41.25 ± 21.75 (20–70)
Anterograde amnesia	2 (5)	n/a	n/a	10
Déjà vu phenomenon	9 (22.5)	20.25 ± 11.17 (2–30)	n/a	30.56 ± 14.24 (10–50)
Expressive dysphasia	5 (12.5)	21 ± 15.59 (3–30)	15.6 ± 13.39 (3–30)	47 ± 36.33 (20–100)
Gibberish speaking	2 (5)	16.5 ± 19.09 (3–30)	16.5 ± 19.09 (3–30)	45 ± 35.35 (20–70)
Slowed speech	11 (27,5)	19.75 ± 19.51 (3–60)	18.64 ± 17.44 (2–60)	59.55 ± 39.9 (10–100)
Dyslexia	10 (25)	21.5 ± 944 (10–30)	21.5 ± 17.89 (3–60)	41.5 ± 27.49 (10–100)
Dyscalculia	2 (5)	37.5 ± 31.82 (15–60)	25 ± 28.28 (5–45)	30 ± 28.28 (10–50)
Astereognosis	2 (5)	16.5 ± 19.09 (3–30)	8 ± 9.9 (1–15)	55 ± 63.64 (10–100)
Manual dyspraxia	8 (20)	21.13 ± 20.25 (4–60)	18.75 ± 21.96 (2–60)	46.88 ± 37.51 (10–100)
Right-left confusion	5 (12.5)	18.75 ± 17.97 (5–45)	13.6 ± 17.74 (3–45)	51 ± 45.33 (10–100)
Neglecting hand symptom	2 (5)	17.5 ± 17.68 (5–30)	17.5 ± 17.68 (5–30)	50 ± 28.28 (30–70)

Onset time - aura onset in regard to beginning of headache expressed in minutes; Time duration - expressed in minutes; Frequency - frequency of symptom compared to all experienced auras (expressed in percentages); n/a - not applicable.

Patients with HCD during aura were classified as HCD group. The 18 patients who did not experience HCD during aura were classified as Standard aura group. Comparison of demographic data and features of aura between these groups are shown in Table 4. There was no statistically significant difference in terms of gender, age at the time of examination, age at migraine onset and duration of aura. Also, these two groups did not significantly differ in number of patients who reported numbness in arms, face and lips as symptom during the aura. Frequency of aura per year was significantly higher in HCD group (6.18 ± 3.17 vs. 3.33 ± 2.03, p = 0.003), as well as the number of patients with somatosensory symptoms during aura (77.3% vs. 38.9%, p = 0.014). Also, HCD group had significantly more patients who reported numbness in hands, tongue and legs compared to Standard aura group.

**Table 4:** Comparison of demographic data and aura features between HCD group and standard aura group

**Demographic data, aura characteristics**	**HCD group (n = 22)**	**Standard aura group (n = 18)**	**Statistics**
Gender – girls (%)	10 (45.5)	10 (55.6)	p = 0.525
Age of patients, X ± SD, years	16.32 ± 1.98	16.06 ± 2.1	p = 0.687
Age at the time of the onset of migraine, X ± SD, in years	13.18 ± 1.74	12.17 ± 2.97	p = 0.212
Aura duration, X ± SD, in minutes	30.91 ± 20.85	27.5 ± 10.04	p = 1.000
Number of auras per year, X ± SD	6.18 ± 3.17	3.33 ± 2.03	p = 0.003
Somatosensory symptoms (%)	17 (77.3)	7 (38.9)	p = 0.014
Numbness in hands (%)	16 (72.7)	5 (27.8)	p = 0.005
Numbness in arms (%)	9 (40.9)	2 (11.1)	p = 0.073
Numbness of the face and lips (%)	9 (40.9)	3 (16.7)	p = 0.165
Numbness of the tongue (%)	10 (45.5)	1 (5.6)	p = 0.011
Numbness in legs (%)	6 (27.3)	0 (0)	p = 0.024

## Discussion

These are the first detailed nosographic descriptions, to our knowledge, of the symptoms experienced during the aura reported by teenagers who have migraine with aura. Our data show that neurological non-visual symptoms, including HCD, during the aura in teenage migraine are notable. Also, the findings of this study clearly demonstrate the variability of aura symptoms. To our opinion, these are important information for pediatricians and other physicians when facing new cases of migraine with unusual aura.

The most common manifestation of migraine with aura is visual phenomenon [14], reported by all our patients. Besides the simple positive or negative phenomena, high number of patients have also reported some form of complex visual disturbances. The most frequent visual symptom was scintillating scotoma, followed by blurry vision, tunnel vision and zig-zag lines. These symptoms had gradual development which corresponds with typically described migraine aura [13]. High prevalence of blurry vision and tunnel vision (shrinking of visual field) in our patients, not typically considered to be an aura phenomenon of cortical origin, support recent findings towards heterogeneous symptoms of visual aura [15]. Moreover, visual dysgnosia during the aura was frequently reported by our patients in contrast to findings in literature [16]. Color dysgnosia, in terms of this - colors get brighter and patients had difficulties in recognition of color shades, was most commonly reported symptom of visual types of dysgnosia and most frequently experienced symptom in patient auras. This could be explained by the fact that visual auras could arise from the primary visual cortex, as well as from other extrastriate areas (e.g. V2, V3yVP, V3A, and V4v) [17,18]. In the other hand, prosopagnosia was reported in only one patient. This could be due to the fact that this function is localized bilaterally [19].

The second most common type of aura in our group of patients was somatosensory phenomena (60%). The most frequently reported somatosensory symptom was numbness in hand (52.5%), while 15% of the patients reported numbness in legs with “marching” phenomenon, which is in line with previous similar study in adult population with migraine [20]. Interestingly, patients with visual and somatosensory aura mainly reported 5 to 10 minutes delay of beginning of somatosensory symptoms after beginning of visual aura, but also in eight patients somatosensory aura onset occurred at the same time or before visual aura. These findings could indicate multiple origin of CSD in some of patients, which is previously proposed [17,20]. It is noteworthy that all patients had normal imaging examinations that excluded a structural lesion which might either account for or be caused by the hyperexcitability that triggers migraines [21].

Beyond the visual and somatosensory symptoms, phenomena reported during the migraine include misperceptions, impaired gnosis, praxis, and memory [8]. In our study, HCD during the aura were notable. Slowed speech and problems in reading as symptoms during the aura were the most usually reported. Moreover, 36% of teenage migraineurs had some type of dysphasic disturbances during the aura, which is high in comparison to 15% reported by Russell MB and Olesen J [16], but less than in our previous study where dysphasic symptoms were reported by 53% of adults with migraine [11]. Furthermore, in teenagers with migraine symptoms of expressive dysphasia were less common (12%) in comparison to adult’s migraine [14]. We can only speculate that in teenage migraineurs CSD rarely reach Broca's region. Also, in our opinion, it is very important to focus on the diversity of dysphasic presentation among migraineurs in further investigations.

One of ten of our patients had memory disturbances during the aura, such as difficulties in remembering the events or more frequently in recalling past events. In patients who had these symptoms every second migraine aura was accompanied by retrograde amnesia. Memory processes involve regions in the medial temporal lobes including the hippocampus, which may be implicated during CSD [8]. Déjà vu phenomenon was reported by 22.5% of our patients, mostly in one third of their auras. Also, our patients reported difficulties in calculating, naming, performing precise movements with hands, orienting in space, recognizing objects by touch, as well as neglecting hand symptom. Frequency of all these HCD occurrences point out the variety of possibilities of CSD propagating through the cortex [8,21].

Further, we compared two subgroups of patients who experienced one or more HCD symptoms during the aura (HCD group) and those who did not experienced HCD (Standard aura group). Main findings were that these subgroups did not differ in terms of gender, age of patients, age at the time of the onset of migraine, aura duration and location of the beginning of visual disturbances in the visual field. This is in line with findings in adult migraine population, except for aura duration, where migraineurs with HCD during the aura had significantly longer duration of aura [11]. Also, HCD group has significantly higher number of auras per year compared to Standard aura group. We can only speculate that this could be due to networks immaturity in teenagers with migraine, which is demonstrated in adolescents [22-24]. Also, we found higher prevalence of somatosensory symptoms in HCD group compared to Standard aura group. Furthermore, we can assume that wideness of somatosensory cortex affected region and CSD intake of secondary somatosensory regions is linked to the number and types of HCD, because the somatosensory cortex plays major importance in multisensory integration processes [25-27].

It should be also mentioned that we had ten patients whose migraine aura proceeded after onset of headache for some period of time. This finding could be of interest for further investigation and more deeply understanding of aura features influence on quality of pain.

The main limitations of our study are the facts that data were collected between attacks and that HCD were determined by patients’ reports. Nevertheless, as the majority of our patients experienced more than 10 auras with a monthly appearance of aura, we could suppose that their descriptions are relevant. Although complex or less clear cognitive changes are particularly susceptible to recall biases, the results clearly indicate that certain cortical dysfunctions must be largely underestimated in migraine because specific questioning is not part of the routine clinical evaluation.

## Conclusions

The analysis of the present cases and general experience [8,11,16] indicates that aura symptoms, regardless of their form, vary to a great extent in duration and complexity from patient to patient, and also within each individual. Hence, we have concluded from our nosographic analysis of migraine aura in teenagers that higher cortical disturbances should be examined more profound in patients. Also, continuously reporting of aura features in teenagers with migraine with HCD during the aura represent great opportunity for neuroimaging investigation of CSD impact on cortex development in adolescents.

### Consent

Written informed consent was obtained from the patient’s parent for the publication of this report.

## Competing interests

The authors declare that they have no competing interests.

## Authors’ contributions

IP carried out study design, interviews with patients, analysis and interpretation of data and drafted the manuscript. VP participated in data collection. DV participated in patient selection and provided database of her patients. JJ carried out study supervision, provided database of her patients and revised the manuscript for content. All authors read and approved the final manuscript.
